# Concentrated Solution of Quinia

**Published:** 1881-06-20

**Authors:** 


**Concentrated Solution of Quinia.**—Dr. S. W. Reynolds, U. S. A., Fort Brown, Texas, writes the American Journal of Pharmacy: I notice in the March number of the "Journal," page 136, a formula for "Lent's solution of quinia," for hypodermic use.

It is not stated when it was first used, but the formula is almost identical with a solution I first made in May, 1874, for every-day use in giving that remedy in the Post Hospital at Fort Stockton, Texas, (the formula for which I had the honor of furnishing you, with the request that you would publish it for the benefit of the pharmacists, especially in the army, but it was never noticed.)

At the time I constructed my formula, I had never seen one of a strength exceeding 30 grains to f℥i, and even *that* was called a concentrated solution. I have continued using my solution, which I have no difficulty in keeping any required length of time, and there is *no* crystalization except in very cold weather, and then it is redissolved very readily by warming.

My formula is as follows:

℞ Quiniæ sulphat.................................. grs. 480.

Acid. sulphur. dil.................................. fʒ vi to f℥ i.

Glycerinæ.................................. fʒ vi.

Aquæ distill.................................. ad f℥ vi.

Misce sec. art. and filter.

I prepare it *without* heating, and find the fʒvi of dilute acid sufficient in warm weather, but in winter the larger quantity is required to maintain the solution. This solution contains exactly 10 grains in each fʒi, while Lent's is not so strong; in fact, the formula referred to does not state the exact strength.

